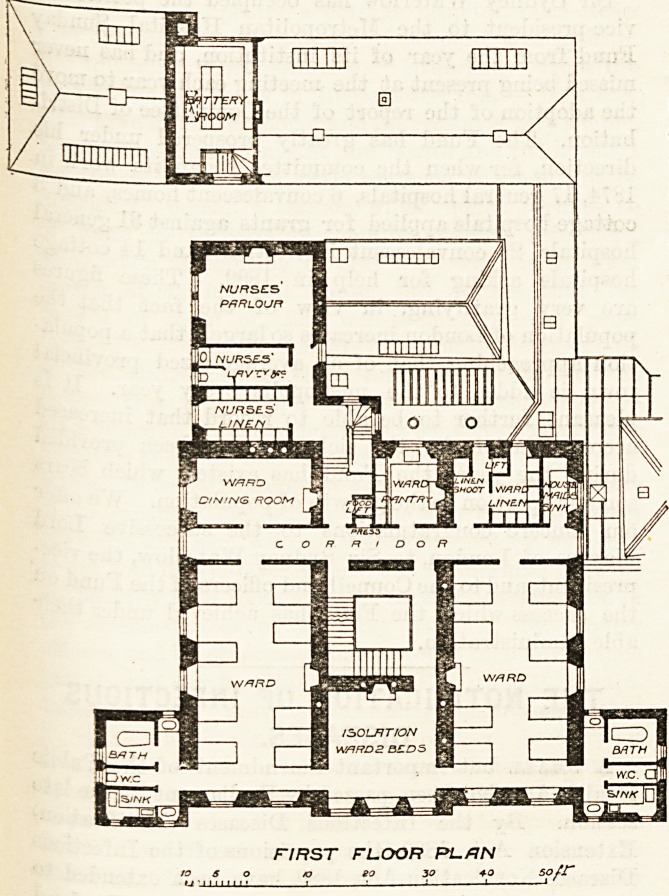# Hospital Construction

**Published:** 1899-09-16

**Authors:** 


### The Institutional Workshop.

### HOSPITAL CONSTRUCTION.

### VICTORIA HOSPITAL, DUNDEE.

This hospital has been converted out of an old mansion. Of the advisableness of such a step we always have grave doubts; but it is sometimes forced on a district by reason of its cheapness as compared with the erection of a purposely built hospital. At any rate, it would be manifestly unfair to follow the same line of criticism in the former case as would be justifiable in the latter.

The north point is not indicated on the plan sent to us, so we are not able to say what the aspect of the main front is; and in the climate of England, still more in that of Scotland, this aspect is a very important thing. The centre of the front part of the building is given up to a fair-sized hall, from which, however, a small room or office for the medical officer has been cut off, and immediately behind the hall is the staircase, which is apparently lighted from the roof. On the left side of the hall on entering is an eight-bedded dormitory. There are four windows in this room—two at one end and two in one side; the other end opens into a corridor, and the other side is enclosed by the hall and staircase. This arrangement is typical of the remaining large dormitories throughout the hospital. The bath-room, closet, and sink are in a small block projecting from one corner of the dormitory; they are separated from the ward by what might be made a ventilating corridor, but this is spoiled by placing a sink under the window at each end. It is a strange thing to have done, as it is unlikely that this part is old, and if new it might just as well have been correctly built. On the opposite side of the hall and staircase are the matron's sitting-room and bedroom and the nurses' dining-room. A similar bath-room block to that already described projects from the matron's sitting-room, but the entrance thereto is, of course, different to that of the ward. In rear of the dormitory, staircase, and nurses' dining-room runs a corridor, which connects them with the ward dining-room, kitchen, servants' hall, &c., and the right-hand end of this corridor gives admission to the cancer ward. This is a five-bedded room, and an isolated single-bedded room adjoins it. Conservatories are attached to one end and part of one side of this ward, and at the back are the pantry, bath-room, and closet. It follows, therefore, that only one side of this important dormitory is free to the open air.

GROUND FLOOR PLAN.

Still further to the rear are the boiler-house, engine-room, and laundry. These are very properly detached from the main building. On the first floor the arrangement of the rooms is similar, except that an eight-bedded dormitory takes the place of the matron's apartments; the cancer ward is not carried up, and a nurses' sitting-room and linen-room are placed over the kitchens. The outer walls are very thick, and advantage has been taken of this to fit up cupboards under the windows. These cupboards are flush with the walls,

FIRST FLOOR PLAN

10 5 0 10 20 30 40 50 ft and are intended for the patients' clothing, which is a very bad plan; the clothing should not be kept in the wards. This space, moreover, would have been the very place in which to put the radiators, hot water being used for warming purposes.

Separate pantries and separate crockery are provided for wards, nurses' dining-room, and servants' hall. The wards are warmed by open fireplaces and hot-water pipes. Electric light is used.

## Figures and Tables

**Figure f1:**
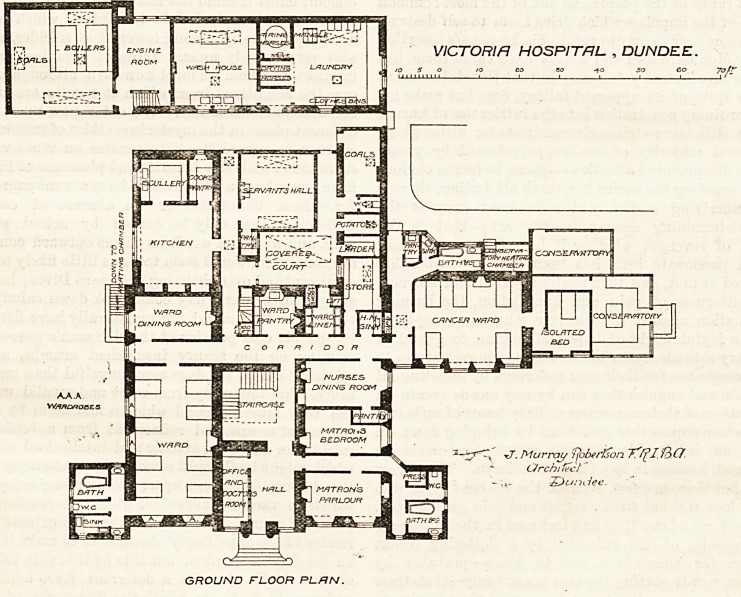


**Figure f2:**